# Knowledge, attitudes and practices towards viral haemorrhagic fevers amongst healthcare workers in urban and rural public healthcare facilities in the N’zérékoré prefecture, Guinea: a cross-sectional study

**DOI:** 10.1186/s12889-020-8433-2

**Published:** 2020-03-06

**Authors:** Manuel Raab, Lisa M. Pfadenhauer, Tamba Jacques Millimouno, Michael Hoelscher, Guenter Froeschl

**Affiliations:** 1 Division of Infectious Diseases and Tropical Medicine, University Hospital (LMU), Leopoldstr. 5, 80802 Munich, Germany; 2 Institute of Medical Informatics, Biometry and Epidemiology, Pettenkofer School of Public Health, Ludwig Maximilian University Munich, Marchioninistr. 15, 81377 Munich, Germany; 3 Department of Disease Surveillance, Agence Nationale de Sécurité Sanitaire (ANSS), Conakry, Guinea

**Keywords:** Viral Haemorrhagic fevers, Ebola, Healthcare workers, Infection prevention and control, Knowledge attitude practice, Guinea, West Africa

## Abstract

**Background:**

The 2013–2016 Ebola epidemic in West Africa began in Guinea’s Forest region, a region now considered to be at high risk for future epidemics of viral haemorrhagic fevers (VHF). Good knowledge, attitudes and practices towards VHF amongst healthcare workers in such regions are a central pillar of infection prevention and control (IPC). To inform future training in IPC, this study assesses the knowledge, attitudes and practices (KAP) towards VHF amongst healthcare workers in public healthcare facilities in the most populated prefecture in Forest Guinea, and compares results from urban and rural areas.

**Methods:**

In June and July 2019, we interviewed 102 healthcare workers in the main urban and rural public healthcare facilities in the N’zérékoré prefecture in Forest Guinea. We used an interviewer-administered questionnaire adapted from validated KAP surveys.

**Results:**

The great majority of respondents demonstrated good knowledge and favourable attitudes towards VHF. However, respondents reported some gaps in preventive practices such as VHF suspect case detection. They also reported a shortage of protective medical equipment used in everyday clinical work in both urban and rural healthcare facilities and a lack of training in IPC, especially in rural healthcare facilities. However, whether or not healthcare workers had been trained in IPC did not seem to influence their level of KAP towards VHF.

**Conclusions:**

Three years after the end of the Ebola epidemic, our findings suggest that public healthcare facilities in the N’zérékoré prefecture in Forest Guinea still lack essential protective equipment and some practical training in VHF suspect case detection. To minimize the risk of future VHF epidemics and improve management of outbreaks of infectious diseases in the region, current efforts to strengthen the public healthcare system in Guinea should encompass questions of supply and IPC training.

## Background

Viral haemorrhagic fevers (VHF) are febrile illnesses caused by distinct families of RNA viruses. The main VHF are Ebola virus disease (EVD), Marburg virus disease (MVD), yellow fever (YF), dengue, Lassa fever (LF), Crimean-Congo haemorrhagic fever (CCHF) and Rift Valley fever (RVF) [1]. Human infection occurs through contact with animal-vectors or other infected humans. Patients may present a wide range of symptoms but VHF usually cause high fever, gastrointestinal symptoms and sometimes bleeding [2]. Clinical outcome depends on the type of viral infection: some VHF only lead to mild illness while others cause life-threatening conditions. Many VHF are highly contagious after an incubation period of up to 21 days, infectiousness of patients usually starting with the onset of symptoms [3]. Thus, VHF outbreaks go hand in hand with a high risk of nosocomial and occupational infection [4].

The largest VHF outbreak to date - caused by *Zaire stain ebolavirus* - occurred in West Africa from 2013 to 2016. It mainly affected Liberia, Sierra Leone and Guinea with a total of more than 28,000 cases [5]. Until March 2015, healthcare workers (HCW) accounted for 3.9% of reported cases [6]. In the current EVD outbreak in the Democratic Republic of the Congo (DRC), 5% of infected cases have been HCW as per august 2019 [7]. High rates of occupational infection are not only due to the contagiousness of certain VHF, but also to increased exposure due to non-recognition of suspect cases, limited and incorrect use of personal protective equipment (PPE), shortcomings in general hygiene practices and infrastructural deficiencies in healthcare facilities [4]. Improvements in those areas have been linked to a decrease in occupational infections during VHF outbreaks [6, 8, 9]. Since HCW are on the frontline of outbreaks, their knowledge, attitudes and practices (KAP) form a central pillar of outbreak preparedness and infection prevention and control (IPC).

Bordering Sierra Leone, Liberia and Ivory Coast, Forest Guinea is one of the four geographic regions of Guinea in the southeast of the country. It was at the epicentre of the 2013–2016 Ebola outbreak and is considered one of the few African viral “hotspots” at high risk for VHF outbreaks, not only for EVD but also for MVD, LF and CCHF [10]. Recently, the region was the site of discovery of *Bombali ebolavirus* in bats and Lassa virus in rodents [11, 12]. To identify misunderstandings, misconceptions and risk practices in HCW relating to EVD, KAP towards EVD amongst HCW have been assessed in parts of Guinea during the EVD outbreak [13–15]: two KAP studies conducted in Guinea’s capital concluded a low knowledge, negative attitudes and practices of EVD in HCW [13, 14]. Another study found that the KAP of EVD suspect cases in HCW were insufficient in regions less affected by EVD [15].

Particularly Guinea’s rural regions have been suffering from a shortage of qualified HCW, especially physicians and nurses [16]. Because of the country’s civil servant system, HCW in rural areas only serve a minimum amount of time before being promoted as civil servants in urban areas, leading to a higher turnover of the health workforce in rural regions. Furthermore, as most HCW receive small salaries, they are often dependent on other sources of income which are mainly unavailable in rural regions. Reforms which hoped to increase workforce retention and professional quality in rural regions after the EVD epidemic have been looked at with scepticism, partly because adequate educational development of HCW in those regions is not ensured [17]. This suggests that HCW practicing in rural regions might be less knowledgeable of VHF and also practice protective measures with a lower frequency.

Assessments of the KAP in HCW as part of epidemic preparedness are important in regions with elevated outbreak risk in order to identify existing gaps and improve occupational safety and viral surveillance. To our current knowledge, no KAP study towards VHF amongst HCW has been conducted in Guinea, nor has any KAP study compared urban and rural HCW respectively in Guinea. The mentioned KAP studies in Guinea focussed on EVD only as part of the outbreak response and not on VHF in general. Furthermore, Forest Guinea as high risk region has never received particular attention as KAP studies were more concentrated in the capital. Our study fills this gap by assessing KAP towards VHF in HCW in urban and rural areas in the N’zérékoré prefecture in Forest Guinea. It informs future training in IPC for HCW in that region and allows to reflect on observed similarities and differences in the knowledge, attitudes and practices towards VHF in HCW practicing either in urban or in rural healthcare facilities.

## Methods

### Setting and study sites

Our study was conducted in June and July 2019 in N’zérékoré, prefecture. N’zérékoré is Forest Guinea’s most populated prefecture and is also the name of the prefecture’s capital city where the largest regional referral hospital of Forest Guinea is situated [18]. Apart from the hospital, 19 health centres are distributed in urban and rural areas to ensure a wide coverage of primary care; numerous small health posts are found in the most remote areas and villages [19]. These are mostly unequipped facilities that provide limited services to the population [20]. As study sites we selected the regional hospital and all 19 main health centres of the prefecture - seven in the urban region and twelve in the rural sub-prefectures. Health posts were not included in our study.

### Respondents

In total, we interviewed 102 HCW. Respondents comprised physicians, nurses, midwives, technical assistants, laboratory staff, pharmacists, non-medical doctors and medical and nurse students. Respondents were selected based on a convenience sample of HCW present during our visits to healthcare facilities. Health centres were visited once for interviews; interviews at the regional hospital were conducted on several days. At each of the healthcare facilities, the director or acting director gave permission to interview HCW and recommended in which order to interview HCW from different services to leave daily routine as undisturbed as possible. We approached respondents based on those recommendations.

### Instruments and scoring system

The questionnaire was developed based on existing KAP studies of EVD from Nigeria and Guinea (view supplementary material). Sections on *knowledge* and *practices* were adapted from Oladimedij et al. [21]. Questions on *knowledge* were adjusted to VHF in general rather than EVD only. The scoring system, which is well-explained elsewhere, was maintained [21]. In short, if three or more out of five questions on incubation period, infectivity, transmission, symptoms and disposal of infected corpse were answered correctly or with a sufficient number of correct answers, knowledge on VHF was scored as “good”. In addition, a list of nine diseases was read out loud to respondents and they were asked to identify the ones that were VHF. This question was not included in the knowledge score. We also added an open-ended question in which respondents were asked which VHF suspect case definition they applied in their daily clinical practice.

Questions on *practices* were adjusted to better fit the Guinean post-Ebola context. Furthermore, the original binary structure of possible answers was changed to a five-point Likert scale to reflect frequency of practices. Three sections - each consisting of nine questions - measured the frequency of practices in general precautions, VHF suspect case identification and VHF suspect case management. The answer “never” received one point, “rarely” two points, “sometimes” three points, “often” four points and “always” five points. An additional possibility “not applicable” was added if HCW felt the demanded practice did not match their clinical responsibility. For questions involving *practices* with clinical materials, a possible answer was added for “material unavailable”. Our scoring system for *practices* deviated from the original version. The mean for each section was calculated and a minimum of four was considered as indicating good practice. Respondents had to reach a good practice score in each section to reach overall good practices. The items where the answers “not applicable” or “material unavailable” were given were excluded from the calculation of the practice score.

The section on *attitudes* was adapted from Jalloh et al. [22]. Questions in this section were adjusted to VHF and to the clinical reality of HCW as respondents. Respondents were not scored in the attitudes section and we did not classify certain attitudes as “discriminatory”.

The language of the questionnaire was French. The original versions in English underwent a forward and back translation by native speakers acquainted with the necessary terminology. In contrast to the reference works of the source questionnaires, our questionnaire was fully interviewer-administered. This was due to the simple reason that overall literacy in French is estimated to be as low as 27% in the N’zérékoré prefecture, leaving even some university graduates with difficulties to read French [23]. Two healthcare professionals from the N’zérékoré prefecture and three graduate students in medicine and pharmacology from N’zérékoré public university were trained in the research protocol and acted as interviewers.

### Statistical analysis

Data was analysed using IBM SPSS 25. Descriptive statistics were generated and proportions were compared using Pearson’s-Chi^2^ Test and Exact Fisher Test. Statistical significance was determined at *p* ≤ 0.05.

### Ethical considerations

The study protocol was approved by the local health authorities, the Guinean National Committee for Research in Health (opinion number 82/CNERS/19) and the Ethics Committee for Medical Research at the Ludwig-Maximilians-Universität (LMU), Munich, Germany (opinion number 18–834). Written informed consent was obtained from all respondents.

## Results

Of the selected healthcare facilities (*n* = 19), one urban and two rural health centres did not participate in the study because insufficient staff was present on the day of our visit. Of all 102 respondents, 58.8% worked in urban healthcare facilities, 41.2% in rural regions of the prefecture; 52% were male. The two most frequently reported professional positions amongst respondents were technical assistant (30.4%) or state registered nurse (22.5%) and the majority (75.5%) had been working in the healthcare facility for more than 1 year. Of those working for more than 1 year, 29.9% had not yet received any training in IPC (view Table 1). In total, 30.4% of all respondents claimed to have never received such training. Significantly more HCW in rural than urban healthcare facilities of the prefecture lacked IPC training (42.9% vs. 21.7%; *p* = 0.029). Amongst those were technical assistants in both rural (58.3%) and urban healthcare facilities (42.1%) and a significantly higher proportion of state nurses in rural than urban healthcare facilities (41.7% vs. 0.0%; *p* = 0.037).

**Table 1:** General Characteristics of Respondents and IPC Training Status by Region

Characteristic	Whole Prefecture	Urban		Rural		*p* value
Number of Participants – n/N (%)	102	60/102 (58.8)		42/102 (41.2)		
Median Age (IQR)	31 (27–38)	32 (27–40)		31 (27–35)		0.69
Gender						0.23
Female - n/N (%)	53/102 (52.0)	28/60 (46.7)		25/42 (59.5)		
Male - n/N (%)	49/102 (48.0)	32/60 (53.3)		17/42 (40.5)		
Duration of Employment in Facility						0.47
0–3 months - n/N (%)	12/102 (11.8)	7/60 (11.7)		5/42 (11.9)		
> 3–12 months - n/N (%)	13/102 (12.7)	6/60 (10.0)		7/42 (16.7)		
> 1 years - n/N (%)	77/102 (75.5)	47/60 (78.3)		30/42 (71.4)		
AND without IPC training	23/77 (29.9)	8/47 (17.0)		15/30 (50.0)		0.003
Working w/o IPC training - n/N (%)	31/102 (30.4)	13/60 (21.7)		18/42 (42.9)		0.029
Professional Position		with IPC	w/o IPC	with IPC	w/o IPC	
State Nurse - n/N (%)	23/102 (22.5)	11/11 (100.0)	0/11 (0.0)	7/12 (58.3)	5/12 (41.7)	0.037
Contractual Nurse - n/N (%)	7/102 (6.9)	5/6 (83.3)	1/6 (16.7)	1/1 (100.0)	0/1 (0.0)	1.00
Student Nurse - n/N (%)	4/102 (3.9)	1/2 (50.0)	1/2 (50.0)	2/2 (100.0)	0/2 (0.0)	1.00
State Physician - n/N (%)	5/102 (4.9)	2/3 (66.7)	1/3 (33.3)	1/2 (50.0)	1/2 (50.0)	1.00
Contractual Physician - n/N (%)	7/102 (6.9)	6/7 (85.7)	1/7 (14.3)	0/0 (0.0)	0/0 (0.0)	
Doctor in different field - n/N (%)	3/102 (2.9)	1/1 (100.0)	0/1 (0.0)	1/2 (50.0)	1/2 (50.0)	1.00
Medical Student - n/N (%)	6/102 (5.9)	6/6 (100.0)	0/6 (0.0)	0/0 (0.0)	0/0 (0.0)	
Technical Assistant (incl. Laboratory) - n/N (%)	31/102 (30.4)	11/19 (57.9)	8/19 (42.1)	5/12 (41.7)	7/12 (58.3)	0.47
Midwife - n/N (%)	14/102 (13.7)	4/5 (80.0)	1/5 (20.0)	6/9 (66.7)	3/9 (33.3)	1.00
Other - n/N (%)	2/102 (2.0)	0/0 (0.0)	0/0 (0.0)	1/2 (50.0)	1/2 (50.0)	

When asked to identify several diseases as VHF, all respondents knew that EVD was a VHF, 85.3% identified LF and 72.5% CCHF correctly as VHF (view Table 2). Only 35.3% knew about MVD and 32.4% about RVF. Overall knowledge on VHF was good amongst 99.0% of all interviewed HCW. Mean knowledge score was 4.3 (SD 0.7) for urban healthcare facilities and 4.2. (SD 0.8) for rural healthcare facilities. Eighty-nine percent of all respondents knew the correct incubation period, 61.8% knew the beginning of infectiousness by clinical signs in patients, 96.1% identified a sufficient amount of modes of transmission, 94.1% cited a sufficient amount of the most frequent initial symptoms and 88.2% knew the correct way to dispose of an infected corpse. We found no significant difference between urban and rural areas regarding knowledge of VHF,

**Table 2:** Summary of Knowledge, Attitudes and Practices, frequency of applied suspect case definitions and availability of materials

	Whole Prefecture	Urban *N* = 60	Rural *N* = 42	*p* value
Number of Participants – n/N (%)	102	60/102 (58.8)	42/102 (41.2)	
Knowledge				
Mean knowledge score max. 5 (SD)	4.3 (0.7)	4.3 (0.7)	4.2 (0.8)	0.52
Overall good knowledge (≥3) - n/N (%)	101/102 (99.0)	60/60 (100.0)	41/42 (97.6)	0.41
Knew correct incubation period - n/N (%)	89/102 (87.3)	54/60 (90.0)	35/42 (83.3)	0.37
Knew the beginning of infectiousness - n/N (%)	63/102 (61.8)	36/60 (60.0)	27/42 (64.3)	0.69
Knew sufficient modes of transmission - n/N (%)	98/102 (96.1)	58/60 (96.7)	40/42 (95.2)	1.00
Knew sufficient initial symptoms - n/N (%)	96/102 (94.1)	56/60 (93.3)	40/42 (95.2)	1.00
Knew correct way to dispose corpse - n/N (%)	90/102 (88.2)	54/102 (90.0)	36/42 (85.7)	0.55
Identified EVD as VHF- n/N (%)	102/102 (100.0)	60/60 (100.0)	42/42 (100.0)	–
Identified LF as VHF- n/N (%)	87/102 (85.3)	49/60 (81.7)	38/42 (90.4)	0.27
Identified CCHF as VHF- n/N (%)	74/102 (72.5)	41/60 (68.3)	33/42 (78.8)	0.27
Identified MVD as VHF- n/N (%)	36/102 (35.3)	23/60 (38.3)	13/42 (31.0)	0.53
Identified RVF as VHF- n/N (%)	33/102 (32.4)	14/60 (23.3)	19/42 (45.2)	0.031
VHF suspect case definition in clinical practice				
Fever and unspecific haemorrhage - n/N (%)	57/102 (55.9)	38/60 (63.3)	19/42 (45.2)	
Fever and specific haemorrhage - n/N (%)	22/102 (21.6)	12/60 (20.0)	10/42 (23.8)	
Fever and general symptoms - n/N (%)	10/102 (9.8)	7/60 (11.7)	3/42 (7.1)	
Other - n/N (%)	8/102 (7.8)	2/60 (3.3)	6/42 (14.3)	
No idea - n/N (%)	5/102 (4.9)	1/60 (1.7)	4/42 (9.5)	
Attitudes				
Would receive survivor as patient - n/N (%)	99/102 (97.1)	57/60 (95.0)	42/42 (100.0)	0.51
Would welcome survivor in community - n/N (%)	99/102 (97.1)	59/60 (98.3)	40/42 (95.2)	0.71
Believed HCW survivor still posed risk to facility - n/N (%)	21/102 (20.6)	8/60 (13.3)	13/42 (31.0)	0.006
Would accept approved vaccine for oneself - n/N (%)	101/102 (99.0)	59/60 (98.3)	42/42 (100.0)	1.00
Would accept approved vaccine for child - n/N (%)	99/102 (97.1)	59/60 (98.3)	40/42 (95.2)	0.17
Would accept experimental treatment for oneself - n/N (%)	41/102 (40.2)	16/60 (26.7)	25/42 (59.5)	< 0.001
Would accept experimental treatment for parent - n/N (%)	38/102 (37.3)	14/60 (23.3)	22/42 (52.4)	0.001
Practices				
Overall good practices - n/N (%)	61/101 (60.4)	33/60 (55.0)	28/41 (66.7)	0.22
Good general precautions - n/N (%)	91/101 (90.1)	50/60 (83.3)	41/41 (100.0)	0.005
Mean general precautions score max. 5 (SD)	4.6 (0.4)	4.4 (0.4)	4.8 (0.2)	< 0.001
Good suspect case identification - n/N (%)	69/97 (71.1)	43/59 (72.9)	26/38 (68.4)	0.65
Mean suspect case identification score max.5 (SD)	4.3 (0.6)	4.3 (0.6)	4.3 (0.6)	0.68
Good suspect case management - n/N (%)	78/90 (86.7)	49/58 (84.5)	29/32 (90.6)	0.53
Mean suspect case management score max. 5 (SD)	4.6 (0.6)	4.6 (0.6)	4.6 (0.5)	0.87
Availability of materials				
Gloves unavailable - n/N (%)	8/102 (7.8)	7/60 (11.7)	1/42 (2.4)	0.14
Masks unavailable - n/N (%)	11/102 (10.8)	10/60 (16.7)	1/42 (2.4)	0.025
Goggles unavailable - n/N (%)	17/102 (16.7)	11/60 (18.3)	6/42 (14.3)	0.79
Infrared thermometer unavailable - n/N (%)	4/102 (3.9)	4/60 (6.7)	0/42 (0.0)	0.14
PPE unavailable - n/N (%)	12/102 (11.8)	12/60 (20.0)	0/42 (0.0)	0.003

When asked which VHF suspect case definition HCW used in their clinical practice, the majority (77.4%) reported to be using a suspect case definition in which the presence of haemorrhage was mandatory. For 55.8% the presence of any haemorrhagic sign coupled with fever was defined a VHF suspect case whereas for 21.6%, only patients with certain haemorrhagic signs such as epistaxis or bloody diarrhoea coupled with fever were considered suspect cases. In 9.8% of cases, respondents used a suspect case definition based on fever and general symptoms.

Almost all HCW (97.1%) held the *attitude* that they would receive a VHF survivor as patient or welcome a VHF survivor back in their community. However, 20.6% believed that a VHF survivor who was also a HCW still posed a risk to their healthcare facility. This was especially the case amongst HCW from rural facilities as compared to urban facilities (31.0% vs 13.3%; *p* = 0.006). When prompted this question, a large number of respondents shared their doubts about their answer, as they said they were unsure about the current scientific consensus regarding lasting viral presence in certain body fluids of EVD survivors. While the large majority of HCW said they would accept an approved vaccine for themselves (99.0%) or their children (97.1%), there was more uncertainty regarding the readiness to receive experimental drugs in case of a VHF infection. Only 40.2% thought they would accept an experimental drug for themselves and 37.3% would accept this for their parents. Here, respondents from rural facilities were more likely to accept experimental treatment for themselves (59.5% vs. 26.7%; *p* < 0.001) and their children (52.4% vs. 23.3%; *p* = 0.001). Otherwise, there was no significant difference in attitudes between HCW from urban and rural regions.

60.4% of interviewed HCW had overall good practices. The items where the answers “not applicable” or “material unavailable” were given were excluded from the calculation of the practice score. 90.1% had good practices in general precautions, 71.1% in suspect case identification and 86.7% in suspect case management. Regarding the lower scores in suspect case identification, respondents reported a low frequency of two particular practices: 50 % (39/78) of respondents claimed they would sometimes or less frequently rule out VHF in patients when it was actually considered necessary and 32.7% (17/52) responded that they never had access to a special triage area for VHF suspect cases.

Similarly, as with VHF knowledge, there was no considerable overall difference in *practices* between respondents from urban and rural health centres even though HCW from rural areas scored slightly higher in general precautionary practices (4.8 vs 4.4, *p* < 0.001). Furthermore, answers to whether HCW would perform a physical exam with a VHF suspect case as part of their suspect case management varied greatly. Of those who found the practice applicable to their clinical situation, 50% (28/56) reported to always perform a physical examination on VHF suspect cases while 36.5% (19/56) said they never practiced a physical examination on a VHF suspect case.

Several respondents did not apply a certain practice because they claimed some materials were lacking. 7.8% of respondents claimed that they did not have any gloves available to follow general precautions in routine practice. To 10.8%, masks were unavailable and 16.7% reported that there were no goggles. Four HCW (3.9%) reported that an infrared thermometer to measure temperature of a suspect case without touching the patient was missing and twelve HCW from urban healthcare facilities (20.0%; *p* = 0.003) claimed that PPE was unavailable to them.

Whether or not HCW had received IPC training did not seem to influence their level of knowledge and their practices of VHF (view Table 3). All respondents who received IPC training had good knowledge of VHF and only one HCW without IPC training received a bad knowledge score (*p* = 0.30). Similarly, both IPC-trained and non-trained HCW had an equal percentage of respondents with good preventive practices (*p* = 1.00).

**Table 3:** Knowledge and Practices by IPC Training

	Received IPC training	Did not receive IPC Training	*p* value
Overall good VHF knowledge	71/71 (100.0)	30/31 (96.8)	0.30
Overall good preventive practices	43/71 (60.6)	18/30 (60.0)	1.00

## Discussion

This is the first KAP study on VHF in Forest Guinea. The study shows that HCW practicing in both urban and rural areas possess a very good knowledge of VHF and would accept vaccines once approved and available. Our data suggests that knowledge on VHF is not a consequence of IPC trainings. Rather, we believe that it is a general consequence of the heightened awareness for VHF that the recent EVD outbreak has created in the country. During our study, HCW consistently emphasized how the EVD epidemic had sharpened their knowledge and practice towards EVD because it had dominated their daily lives in healthcare facilities for so long.

We found some deficiencies in practices amongst many of our respondents in both urban and rural healthcare facilities. Especially the fact that HCW did not consistently rule out VHF in patients when they presented a certain range of symptoms – e.g. through the clinical exploration of more likely causes for the symptoms or through recommended laboratory tests for VHF – is an important finding. It points to a reluctance of HCW to signal VHF suspect cases even though they may fit suspect case definition criteria. This may seriously jeopardize routine VHF screening in clinical practice.

Furthermore, the large majority of HCW reported to be using a VHF suspect case definition based on the presence of haemorrhagic signs in patients. While this reflects WHO recommendations for integrated disease surveillance in African countries, such case definitions risk to miss VHF index cases since visible haemorrhage is only an infrequent and late clinical sign of infection, at least in EVD [24].

As previous Ebola-related KAP surveys amongst HCW in Guinea during the EVD epidemic, we identified IPC training needs with regards to reported IPC training status and the described deficiencies in practices. The EVD epidemic has generated widespread awareness of the disease and left many HCW in the country with IPC training who had been without before [25–27]. Despite the increased post-Ebola efforts to improve IPC performance and VHF awareness in healthcare facilities, our study hints at a gap in IPC training in the N’zérékoré region, especially in rural areas. Similar findings have been reported in other West African countries [21, 28]. Surprisingly, this gap does not seem to translate into a lack of knowledge or deficient practices towards VHF. A recent study on IPC in the DRC suggested that a higher quantity of HCW with IPC training does not increase IPC performance of healthcare facilities [29]. We found that rural areas where significantly less HCW had received IPC training than in the urban areas reported higher scores on certain topics regarding *practices*. Even though our data cannot explain this finding, informal conversations with HCW have indicated that HCW in rural areas feel strongly responsible for preventing any future outbreaks in rural regions since such events would stigmatize “their” region as sanitary problem area once more. This underscores that quantity itself may not suffice when aiming to improve IPC performance through trainings. Nevertheless, future index cases of VHF outbreaks are likely to appear in Forest Guinea and we believe that the above reported deficiencies in practices regarding VHF suspect case detection in both urban and rural areas have to be tackled, possibly through improving the quality of IPC trainings.

Last, several respondents in our study reported the absence of important basic protective materials, such as gloves, masks and goggles– a fact already well documented during the 2013–2016 EVD outbreak [30]. PPE seemed to be less available in urban than in rural healthcare facilities. According to some HCW, this was due to a temporary distribution problem since specialized treatment centres for infectious diseases have opened in 2018 in urban areas. These centres stockpiled specialized gear such as PPE for urban healthcare facilities in preparation for future outbreaks.

Our study has two main limitations. First, our results are based on a non-probabilistic sampling strategy. Therefore, our comparison between urban and rural healthcare facilities has to be interpreted with caution. Financial and temporal restrictions as well as an unknown target population size prevented us from employing a probabilistic sampling strategy. Our stratification into urban and rural subgroups was thus not based on prior knowledge of population sizes. At the time of our study, 432 HCW were employed in the N’zérékoré prefecture according to the local health authorities but rural and urban proportions were unknown. The official numbers, however, rarely reflect the reality in healthcare facilities: a recent study on Guinean health workforce has noted absenteeism rates between 39 and 41% [17]. The same study found that (graduate) students and other volunteers– not represented in official numbers - possibly make up for the absence of civil servants. Since this part of the health workforce executes similar tasks as officially registered HCW - such as triage, diagnostics and therapy - they are exposed to the same occupational risks. Assessing their knowledge attitudes and practices towards VHF was considered of equal relevance for this study which is reflected in the composition of our study population.

Second, the reported deficiencies in protective practices are possibly bigger than our study shows. As the questionnaire was interviewer-administered and not accompanied by a verification process, over-reporting of practices due to social desirability may have occurred. For instance, a recent study in the N’zérékoré regional hospital showed that the great majority of VHF suspect cases are usually not recognized by HCW [31] even though 71.1% of HCW in our study reported good suspect case identification practices.

Despite these limitations, our study provides insight into existing shortcomings in practices amongst HCW in a remote region of Guinea where access to rural areas is often difficult for researchers.

## Conclusion

Good knowledge, attitudes and practices towards VHF amongst healthcare workers form a central pillar of infection control and prevention in regions at risk for VHF outbreaks. Our study – conducted in urban and rural public healthcare facilities in the former epicentre of the 2013–2016 EVD outbreak in Guinea– showed that HCW possess a good knowledge of VHF but maintain some deficiencies in practices regarding the management of VHF. Whether or not HCW were trained in IPC did not influence their KAP. Thus, while the study highlighted deficiencies in some crucial preventive practices such as VHF suspect case detection, the need for more training in IPC is not self-evident. Rather, future IPC trainings in Forest Guinea should ensure that a certain quality in IPC is met and maintained by healthcare facilities, especially regarding future VHF signal case detection and outbreak prevention. This study further hinted to a shortage in the provision of general protective materials and PPE. Future efforts should thus also only aim to improve the availability protective gear in healthcare facilities.

## Supplementary information

**Additional file 1.**

## Data Availability

Due to Guinean national regulations, data cannot be made freely accessible. Dataset can be provided, however, upon well-reasoned request and upon clearance by the involved Guinean authorities. For requests, please contact the corresponding author.

## Abbreviations

CCHF: Crimean-Congo haemorrhagic fever
DRC: Democratic Republic of the Congo
EVD: Ebola virus disease
HCW: Healthcare worker
IPC: Infection prevention and control
KAP: Knowledge, attitudes and practices
LF: Lassa fever
MVD: Marburg virus disease
PPE: Personal protective equipment
RVF: Rift valley fever
VHF: Viral haemorrhagic fever
YF: Yellow fever
