# Supplementary material for: Knowledge, attitudes and practices towards viral haemorrhagic fevers amongst healthcare workers in urban and rural public healthcare facilities in the N’zérékoré prefecture, Guinea: a cross-sectional study
Source: BMC Public Health. 2020 Mar 6;20:296. doi: 10.1186/s12889-020-8433-2 (PMC7059383; doi:10.1186/s12889-020-8433-2)
Supplement: Supplementary file 1 — Additional file 1. [file 12889_2020_8433_MOESM1_ESM.pdf]

Knowledge, Attitudes and Practices towards Viral Hemorrhagic Fevers among healthcare professionals in the Hôpital Régional de N'Zérékoré (HRN'Z)

Interviewer administered questionnaire
Parts "Knowledge" and "Practices" modified after Oladimeji et al. 2015 (1)
Part "Attitudes" modified after Jalloh et. al. 2017 (2)

Number of Questionnaire:

1) General Information

1. Gender: Male Female

2. Age (in years):

3. How long have you been working at the HRN'Z?
   a) Less than 3 months
   b) Between 3 months and 1 year
   c) Between 1 year and 3 years
   d) More than 3 years

4. What is your current professional position at the HRN'Z?
   a) State Nurse
   b) Contract Nurse
   c) Student Nurse
   d) State Medical Doctor
   e) Contract Medical Doctor
   f) Doctor of a different discipline (for example Biology)
   g) Medical student
   h) Technical Assistant/Laboratory Technician
   i) Midwife
   j) Other (specify):

5. Which medical service are you currently working in (if you are working in several medical services, please indicate the service where you spend most working hours)?
   a) HIV-Treatment Center ("CTA")
   b) Laboratory
   c) Pharmacy
   d) General Medicine (including Diabetology and Cardiology)
   e) Otorhinolaryngology
   f) Surgery
   g) Maternity/Obstetrics
   h) Emergency Room
   i) Dental Care
   j) Ophthalmology
   k) Triage

2) Knowledge

Please answer the following questions regarding your current knowledge and attitudes towards Viral Hemorrhagic Fevers. One answer or multiple answers may be correct. This will be indicated to you by the interviewer.

6. Which definition of a Viral Hemorrhagic Fever suspect case do you use in your clinical practice?

7. According to you, which of the following diseases is a Viral Hemorrhagic Fever (multiple answers are correct)?
   a) HIV/AIDS
   b) Lassa Fever
   c) Typhoid Fever
   d) Ebola Virus Disease
   e) Marburg Virus Disease
   f) Crimean Congo Hemorrhagic Fever
   g) Rift Valley Fever
   h) Meningitis
   i) Acute Fever of unknown origin

8. How long is usually the incubation period of Viral Hemorrhagic Fevers (one answer is correct)?
   a) Between a few hours and 1 day
   b) Between 2 days and 3 weeks
   c) More than 3 weeks
   d) I don't know

9. When does a patient infected with a Viral Hemorrhagic Fever usually become infectious (one answer is correct)?
   a) When he/she is without symptoms
   b) On the 1st day of his/her symptoms
   c) 3 days after the onset of symptoms
   d) I don't know

10. How can Viral Hemorrhagic Fevers be transmitted? (multiple answers are correct)
    a) Through contact with an infected animal
    b) Through direct physical contact with an infected person presenting symptoms of Viral Hemorrhagic Fever infection
    c) Through contact with biological fluids (saliva, urine, stool, blood) from an infected person presenting symptoms of Viral Hemorrhagic Fever infection
    d) Through contact with clothing, beddings and possessions from an infected person presenting symptoms of Viral Hemorrhagic Fever infection
    e) Through participation in the burial of an infected individual

11. Which are the most frequent primary symptoms of Viral Hemorrhagic Fevers (multiple answers are be correct)?
    a) Acute fever
    b) Weight loss
    c) Tiredness
    d) Dizziness
    e) Cardiac arrest
    f) Muscle aches
    g) Loss of strength
    h) Depression

12. How should deceased patients who were infected with a Viral Hemorrhagic Fever be buried (one answer is correct)?
    a) No special measures need to be taken
    b) Through cremation or burial in a sealed bag
    c) A traditional burial is preferable
    d) A medical doctor should examine the corpse to establish the cause of death prior to the burial
    e) I don't know

3) Attitudes

3A: Attitudes towards Viral Hemorrhagic Fever survivors

13. Do you think that a healthcare professional who has survived a Viral Hemorrhagic Fever infection (for example Ebola) and now possesses a certificate from a public healthcare facility stating that he/she has survived the disease, still present a risk of infection for the healthcare professionals of that healthcare facility?
    a) Yes
    b) No
    c) I do not know / I am not sure
    d) I decline to answer

14. If you knew a merchant who has survived a Viral Hemorrhagic Fever infection (for example Ebola) and possessed a certificate from a public healthcare facility stating that he/she has survived the disease, would you be ready to buy vegetables from this merchant?
    a) Yes
    b) No
    c) I do not know / I am not sure
    d) I decline to answer

15. Would you accept that a neighbor who has survived a Viral Hemorrhagic Fever infection (for example Ebola) returns to your community?
    a) Yes
    b) No
    c) I do not know / I am not sure
    e) I decline to answer

3B: Perceptions of VHF vaccine and experimental treatment

16. If there was an approved vaccine capable of preventing a Viral Hemorrhagic Fever (for example Ebola), would you accept to vaccinate yourself?
    a) Yes
    b) No
    c) I do not know / I am not sure
    d) I decline to answer

17. If there was an approved vaccine capable of preventing a Viral Hemorrhagic Fever (for example Ebola), would you accept to vaccinate your children?
    a) Yes
    b) No
    c) Not applicable. I do not have any children
    d) I do not know / I am not sure
    e) I decline to answer

18. Imagine you were infected with a Viral Hemorrhagic Fever (for example Ebola), and there existed a treatment with promising results amongst infected animals and humans but the treatment was not officially approved. Would you accept this treatment?
    a) Yes
    b) No
    c) I do not know / I am not sure
    d) I decline to answer

19. Imagine your relative was infected with a Viral Hemorrhagic Fever (for example Ebola), and there existed a treatment with promising results amongst infected animals and humans but the treatment was not officially approved. Would you accept this treatment for your relative?
    a) Yes
    b) No
    c) I do not know / I am not sure
    d) I decline to answer

4) Practices

Would you please indicate which of the following practices you follow and with which frequency? Certain practices may be followed by all healthcare professionals, others only by a medical doctor, a nurse or a technical assistant. If you do not execute a specific task yourself (for example notifying the authorities of a Viral Hemorrhagic Fever suspect case) and another team member takes care of it (for example the head of service), that means that you, as a member of the service, do not execute that specific task and the correct response is “not applicable”.

1. I take the following precautions in my daily clinical practice…

20. I wash my hands before and after touching a patient	Always<br>Often<br>Sometimes<br>Rarely<br>Never<br>Not applicable
21. I wear gloves	Always<br>Often<br>Sometimes<br>Rarely<br>Never<br>Gloves are not available<br>Not applicable
22. I use a mask when the patient is suspected to have an infectious disease	Always<br>Often<br>Sometimes<br>Rarely<br>Never<br>Masks are not available<br>Not applicable
23. I use protective goggles when a patient is suspected to have an infectious diseases	Always<br>Often<br>Sometimes<br>Rarely<br>Never<br>Goggles are not available<br>Not applicable
24. I avoid recapping of needles	Always<br>Often<br>Sometimes<br>Rarely<br>Never<br>Not applicable
25. I use sharp boxes	Always<br>Often<br>Sometimes<br>Rarely<br>Never<br>Sharp boxes are not available<br>Not applicable

26. I dispose of waste in a safely manner by placing it into the provided waste containers according to the type of waste	Always<br>Often<br>Sometimes<br>Rarely<br>Never<br>Waste containers are not available<br>Not applicable
27. I disinfect medical care equipment before using it on another patient	Always<br>Often<br>Sometimes<br>Rarely<br>Never<br>Disinfectant is not available<br>Not applicable
28. I take other precautions (please specify):	Always<br>Often<br>Sometimes<br>Rarely<br>Never<br>Not applicable

2. To identify Viral Hemorrhagic suspect cases, I execute the following tasks…

29. I measure the temperature of every patient	Always<br>Often<br>Sometimes<br>Rarely<br>Never<br>Thermometer is not available<br>Not applicable
30. I ask the patient whether he/she has a fever	Always<br>Often<br>Sometimes<br>Rarely<br>Never<br>Not applicable
31. I ask the patient if he/she presents symptoms like fever, diarrhea, vomiting, unexplained bleeding, etc.	Always<br>Often<br>Sometimes<br>Rarely<br>Never<br>Not applicable

32. I ask every suspect patient whether he/she has been exposed to sick people with similar symptoms	Always<br>Often<br>Sometimes<br>Rarely<br>Never<br>Not applicable
33. I ask every suspect patient whether he/she has recently travelled to regions with epidemics (for example DRC: Ebola, Nigeria/certain regions of Guinea: Lassa, etc.)	Always<br>Often<br>Sometimes<br>Rarely<br>Never<br>Not applicable
34. I rule out Viral Hemorrhagic Fever as possible cause for the disease, if necessary	Always<br>Often<br>Sometimes<br>Rarely<br>Never<br>Not applicable
35. I have access to a special triage area for febrile patients who fit the Viral Hemorrhagic Fever suspect case definition	Always<br>Often<br>Sometimes<br>Rarely<br>Never<br>Not applicable
36. I draw / order to draw blood for testing Viral Hemorrhagic Fevers	Always<br>Often<br>Sometimes<br>Rarely<br>Never<br>Not applicable
37. I invest more time and effort than usually to identify Viral Hemorrhagic Fever suspect cases (please specify):	Always<br>Often<br>Sometimes<br>Rarely<br>Never<br>Not applicable

3. When I have identified a Viral Hemorrhagic Fever suspect case, I take the following measures…

38. I isolate the patient	Always<br>Often<br>Sometimes<br>Rarely<br>Never<br>Not applicable

39. I keep a security distance of one meter from the patient	Always<br>Often<br>Sometimes<br>Rarely<br>Never<br>Not applicable
40. I take general precautions measures	Always<br>Often<br>Sometimes<br>Rarely<br>Never<br>Not applicable
41. I report the case to the responsible medical doctor	Always<br>Often<br>Sometimes<br>Rarely<br>Never<br>Not applicable
42. I obtain the patient's history of exposure / contact	Always<br>Often<br>Sometimes<br>Rarely<br>Never<br>Not applicable
43. I take the patient's temperature without touching him/her	Always<br>Often<br>Sometimes<br>Rarely<br>Never<br>Thermometer is not available<br>Not applicable
44. I do a physical examination	Always<br>Often<br>Sometimes<br>Rarely<br>Never<br>Not applicable
45. I wear a mask and gloves	Always<br>Often<br>Sometimes<br>Rarely<br>Never<br>Masks and gloves are not available<br>Not applicable

46. I wear full Personal Protective Equipment (PPE)	Always<br>Often<br>Sometimes<br>Rarely<br>Never<br>PPE is not available<br>Not applicable
47. I notify the responsible authorities	Always<br>Often<br>Sometimes<br>Rarely<br>Never<br>Not applicable
48. I refer the patient to the structure of reference (CTEpi)	Always<br>Often<br>Sometimes<br>Rarely<br>Never<br>Not applicable

1. Oladimeji AM, Gidado S, Nguku P, Nwangwu IG, Patil ND, Oladosu F, et al. Ebola virus disease – gaps in knowledge and practice among healthcare workers in Lagos, August 2014. Tropical Medicine & International Health. 2015;20(9):1162–70.

2. Jalloh MF, Sengeh P, Monasch R, Jalloh MB, DeLuca N, Dyson M, et al. National survey of Ebola-related knowledge, attitudes and practices before the outbreak peak in Sierra Leone: August 2014. BMJ Glob Health. 2017;2(4).
